# Impact of Diabetes Type 1 in Children on Autonomic Modulation at Rest and in Response to the Active Orthostatic Test

**DOI:** 10.1371/journal.pone.0164375

**Published:** 2016-10-27

**Authors:** Thais Roque Giacon, Franciele Marques Vanderlei, Diego Giulliano Destro Christofaro, Luiz Carlos Marques Vanderlei

**Affiliations:** 1 Programa de Pós Graduação em Fisioterapia, Faculdade de Ciências e Tecnologia – FCT/UNESP, Presidente Prudente, SP, Brazil; 2 Departamento de Fisioterapia, Faculdade de Ciências e Tecnologia – FCT/UNESP, Presidente Prudente, SP, Brazil; 3 Departamento de Educação Física. Faculdade de Ciências e Tecnologia – FCT/UNESP, Presidente Prudente, SP, Brazil; TNO, NETHERLANDS

## Abstract

**Introduction:**

Cardiovascular autonomic neuropathy is one of the most common complications of diabetes mellitus type 1 (DM1), of which one of the first subclinical manifestations is changes in heart rate variability (HRV). Thus, analysis of HRV associated with the autonomic active orthostatic test is important in this population.

**Objectives:**

To analyze the autonomic modulation responses induced by the implementation of the active orthostatic test, in children with DM1, and study the autonomic modulation by means of HRV indices.

**Method:**

Data of 35 children were analyzed, of both sexes, aged between 7 and 15 years, who were divided into two groups: Diabetic (n = 16) and Control (n = 19). The following variables were collected initially: weight, height, body fat percentage, heart rate, blood pressure and casual blood glucose. Subsequently, for analysis of autonomic modulation, the beat-to-beat heart rate was captured by a heart rate monitor in the supine position for 30 minutes and after 10 minutes standing during performance of the active orthostatic test. HRV indices were calculated in the time and frequency domains. For data analysis, covariance analysis was used to compare groups and ANOVA for repeated measures to compare the effects of the active orthostatic test. These data were adjusted for age, sex, ethnicity, body fat percentage and casual blood glucose, with a 5% significance level.

**Results:**

The results suggested that diabetic children at rest present a decrease in SDNN (50.4 vs. 75.2), rMSSD (38.7 vs 57.6) and LF [ms^2^] (693.6 vs 1874.6). During the active orthostatic test the children in both groups demonstrated a reduction in SDNN, RMSSD and LF [ms^2^] compared to the resting position, and this response was less pronounced in the diabetic group.

**Conclusion:**

We conclude that regardless of age, sex, ethnicity, body fat percentage and casual blood glucose, performing the active orthostatic test promoted increased sympathetic modulation and reduced parasympathetic modulation in both groups, and this response was less pronounced in diabetic children, who presented reduced overall variability and parasympathetic modulation.

## Introduction

Diabetes mellitus (DM) is considered a serious public health problem due to an increase in the prevalence, morbidity and mortality in developed and developing countries [1]. In childhood, DM type 1 (DM1) has been indicated as one of the most common endocrine diseases [2,3], and in the world it is estimated that there are over 500,000 children under 15 who present DM1, while Brazil has 31,000 registered cases of children diagnosed with this pathology [2].

Diabetic autonomic neuropathy (DAN) is one of the most common complications of DM1 and can affect any organ system, however, in general, it initially affects the cardiovascular system, triggering cardiovascular autonomic neuropathy (CAN) [4–6]. In DM1 individuals, autonomic alterations resulting from CAN are diagnosed late, being discovered when the disease is already in advanced and irreversible stages [3], which aggravates the clinical condition and is associated with various complications and consequent increased mortality [4].

One of the first sub-clinical manifestations of CAN can be alterations in heart rate variability (HRV) [3,4], a method that describes the oscillations of RR intervals between consecutive heartbeats, providing information on the autonomic nervous system (ANS) [7–9].

Studies indicate that diabetic children present HRV alterations characterized by reduction in overall variability and parasympathetic activity [10–16], however, these studies do not consider factors that influence the autonomic changes such as sex [17], age [18], ethnicity [19], body fat percentage [20] and casual blood glucose [15].

For a more complete analysis of the ANS, the association of analysis of HRV indices with the realization of autonomic testing can be an effective combination [21]. These tests are based on the application of a stimulus and, sequentially, observation of the physiological response of the target organ to a known autonomic reflex, or by using drugs that interfere directly or indirectly in the activity of the ANS [22].

One physiological autonomic test which is used is the orthostatic test [21,22], which promotes stimulus of ANS characterized by increased sympathetic modulation and vagal inhibition and can be performed actively or passively [23]. The active orthostatic test is a simple assessment when compared to the passive orthostatic test and other autonomic tests, in addition to being a low-cost method [23].

Despite its importance, few studies have evaluated autonomic modulation, associating HRV with autonomic tests in this population. After searching the relevant technical literature, only one study was found using the active orthostatic test in diabetic children [14]. In this study it was observed that the HRV indices followed a pattern of sympathetic activation and vagal withdrawal during the realization of the active orthostatic test, however these changes were similar in DM1 and healthy individuals [14].

In this context, studying the analysis of HRV associated with autonomic tests may help in better understanding the influence of DM1 on ANS, assisting in the evaluation and therapeutic monitoring of these patients.

Thus, in order to add elements to the literature on the above theme, the aim of the present study was to analyze the autonomic modulation response induced by performing the active orthostatic test in children with DM1 and study the autonomic modulation by means of HRV indices in the time and frequency domains, adjusted for sex, age, ethnicity, body fat percentage and casual blood glucose. We hypothesized that the HRV indices in the time and frequency domains and the active orthostatic test responses would be able to identify alterations in the autonomic modulation of children with DM1, independent of the above mentioned confounding factors.

## Methods

### Population

For this study, data from 35 children of both sexes were evaluated, aged between 7 and 15 and divided into two groups: DM1 and control. For the sample size, a sample calculation based on the rMSSD index was carried out. The magnitude assumed a significant difference of 19 milliseconds (ms), considering a standard deviation of 16 ms, with an alpha risk of 5% and beta risk of 80%; the sample size resulted in at least 11 children in each group.

Children who reported any of the following characteristics were excluded: use of medicines that would influence the autonomic activity of the heart, such as propranolol and atropine, infections, metabolic diseases, except DM1, and known cardiorespiratory system diseases. Children who presented RR interval series with errors greater than 5% and those who were overweight or obese were excluded from the study.

The diabetic group was selected from patients enrolled in the Regional Hospital of Presidente Prudente. For this selection, the hospital initially provided 50 telephone contacts of diabetic children, of these, 28 children were excluded either because they could not participate in the study, due to the above inclusion criteria, did not answer the telephone calls or refused to participate in the data collection. Of the 22 remaining children, 3 did not attend the collections and 3 were excluded due to an error greater than 5% in the RR interval series. For formation of the control group, 30 healthy children were recruited from the Sociedade Civil Beneficente LAR Santa Filomena in Presidente Prudente. Of these children, 11 were excluded, 8 for presenting an error greater than 5% in the tracing and 3 who were overweight or obese.

The DM1 group consisted of 16 volunteers with a diagnosis of DM1 (11 boys and 5 girls) and a time of diagnosis of 4.5 ± 2.6 years. The diabetic children were treated with insulin dosages. The control group consisted of 19 apparently healthy children, 12 boys and 7 girls.

The characteristics of the children (age, weight, height, body mass index, body fat percentage, blood pressure, heart rate and casual blood glucose) are presented in Table 1.

**Table 1:** Characterization of the children in the diabetic and control groups, for age, HR, SBP, DBP, height, weight, BMI, percentage body fat and casual blood glucose.

Variable	Diabetes	Control	p-value
**Age (years)**	12.1 (2.1) 11 [9–15]	11.7 (1.6) 12 [7–15]	0.2780
**HR (bpm)**	83.5 (15.7) 81 [60–120]	82.8 (12.5) 85 [62–104]	0.4472
**SBP (mmHg)**	107.1 (9.1) 110 [120–90]	101.6 (11.2) 100 [120–80]	0.0601
**DBP (mmHg)**	65.9 (8.0) 70 [80–50]	65.3 (9.6) 70 [80–40]	0.4180
**Height (meters)**	1.5 (0.2) 1.5 [1.2–1.7]	1.5 (0.1) 1.6 [1.4–1.7]	0.3632
**Weight (Kg)**	44.2 (13.0) 44.2 [23.6–68.8]	47.7 (9.0) 49.3 [32.9–60.2]	0.1710
**BMI (kg.m**^**2**^**)**	18.4 (2.9) 17.8 [14.6–25.2]	19.9 (2.9) 19.9 [15.9–24.9]	0.0551
**Percentage body fat**	20.3 (7.0) 21 [5.5–30]	22.9 (5.5) 24.2 [12.6–32.3]	0.1245
**Casual blood glucose**	195.8 (95.6) 180 [87–488]	100.6 (18.0) 95 [83–156]	0.0001*

Mean (standard deviation); Median [minimum—maximum]; HR = heart rate; SBP = systolic blood pressure; DBP = diastolic blood pressure; BMI = body mass index; % = Percentage.

*p<0.05.

All procedures in this study were approved by the Institutional Ethics Committee (CAAE: 30935414.1.0000.5402/818.293) and the children and those responsible were duly informed about the procedures and objectives of this study, and after agreeing, the responsible adult signed a consent term and the children an assent term, constituting a possible sample.

### Experimental Protocol

The volunteers were evaluated in a room with a temperature between 21 and 23°C and humidity between 40 and 60%. All children were instructed not to drink ANS stimulants, such as coffee, tea and chocolate drinks, for a period of 12 hours prior to the evaluation, and not to perform intense physical activity on the day of the assessments. Before the autonomic assessment the children were instructed to remain silent and awake, spontaneously breathing at rest for 30 minutes in the supine position.

Before the start of the experimental procedure the children were identified by collecting the following information: age, sex, ethnicity, possible symptoms and associated pathologies.

After identification, weight, height, blood pressure, heart rate (HR), body fat percentage and casual blood glucose glycemia were measured. Next, a capture strap was placed at the distal third of the sternum and a heart rate receiver Polar S810i (Polar, Finland) on the wrist, to capture the heart rate beat-to-beat, with the child at rest, breathing spontaneously for 30 minutes in the supine position on a mattress. After the capture of HR at rest, the children performed the active orthostatic test and were then released.

### Cardiovascular Parameter Assessment

Blood pressure was measured indirectly using a stethoscope (Littman, Saint Paul, USA) and an aneroid sphygmomanometer (Welch Allyn, New York, USA) on the left arm of the children, following the criteria established by the VI Brazilian Guidelines on Arterial Hypertension [24]. HR was captured by means of a frequency meter, Polar S810i (Polar Electro, Kempele, Finland), previously validated for capture of RR intervals, as well as for HRV analysis using the interval series obtained [25,26].

### Body Composition Assessment

For the body composition assessment, the weight and height of the children were measured to calculate the body mass index (BMI) and percentage of body fat. Body mass was determined using a digital scale (Welmy R/I200, Brazil) and height by a stadiometer (Sanny, Brazil). From the measurements of weight and height, BMI was calculated using the formula of weight (kilograms) divided by height (meters) squared [27].

The body fat percentage was determined by bioimpedance (Body Fat Analyzer BF 906, Maltron, UK) with the child in the supine position on a nonconductive surface, without contact with metal, the lower limbs were abducted 45° and the upper limbs 30° to avoid contact of the limbs with the trunk.

To perform the analysis, electrodes were placed at the following locations with a minimum distance of 5 cm between them, after cleaning with alcohol: one electrode at the base of the third finger and another just above the wrist joint, near the styloid process of the right hand, as well as at the base of the third toe of the right foot and just above the ankle joint, between the medial and lateral malleolus [28].

### Metabolic Parameter Assessment

The evaluation of casual blood glucose was performed through fingertip puncture using the measurement device OneTouch (Johhson, Argentina) [1]. For this examination there were no dietary restrictions.

### Autonomic Assessment

For autonomic assessment, a capture strap was positioned at the distal third of the sternum of the children and the heart rate receiver, Polar S810i (Polar^®^, Finland), on the wrist [25,29] and HR beat-to-beat was recorded throughout the experimental protocol with a sampling rate of 1000 Hz.

The series of RR intervals was subjected to digital filtering complemented by manual to eliminate premature ectopic beats and RR artifacts and only series with more than 95% sinus beats were included in the study [29,30]. HRV indices in the time and frequency domains were calculated using the *software Kubios HRV* (*version* 2.0) [31].

In the time domain, the SDNN and rMSSD indices were calculated. The SDNN index represents the standard deviation of all RR intervals [7] and the rMSSD the root mean square of the successive differences between adjacent normal RR intervals [7]. For the HRV analysis in the frequency domain the high frequency (HF, 0.15 to 0.4 Hz) and low frequency (LF, 0.04 to 0.15 Hz) spectral components were analyzed in ms^2^ and normalized units, and the ratio of these components (LF/HF ratio). Spectral analysis was calculated using the Fourier Transform algorithm [7].

For analysis of the HRV indices at rest and during the test, a five minute recorded interval was used and verified to contain a minimum of 256 beats.

### Autonomic Test—Active Orthostatic Test

To perform the active orthostatic test the children were instructed to remain lying at rest for 30 minutes, after which they stood up (3 to 4s), remaining in a standing position for 10 minutes. The children were monitored throughout the period [22,32]. The RR interval sequence with greater stability was selected from the initial rest period and during the maneuver for each child [23]

### Data analysis

Descriptive statistics were used to characterize the population and the results presented as mean, standard deviation, median, minimum and maximum values. For comparison of the characteristics of the groups, data normality was determined using the Shapiro-Wilk test. For data with normal distribution the Student t test for unpaired data was used. When normal distribution was not accepted the Mann-Whitney test was used.

To compare the values of HRV indices between the groups at rest, Analysis of Covariance (ANCOVA) was used controlled by the following confounding factors: age, sex, ethnicity, body fat percentage and casual blood glucose. To compare the effects of the active orthostatic test on the HRV of the children with and without diabetes, considering the pre- and post-test moments, Analysis of Variance (ANOVA) for repeated measures was used, adjusted for age, sex, ethnicity, body fat percentage and casual blood glucose. Possible differences were identified by the Bonferroni post-hoc test and the effect size was calculated using Eta-squared.

In all tests, the statistical significance used was 5%. The statistical program was SPSS version 15.0.

## Results

Table 1 (S1 and S2 Files complement) presents the characterization of the volunteers from both groups, considering age, HR, systolic blood pressure (SBP), diastolic blood pressure (DBP), height, weight, BMI, body fat percentage and casual blood glucose. No statistically significant differences were observed between the groups for these variables, except for casual blood glucose (p = 0.0001).

Table 2 (S3 and S4 Files complement) shows the comparisons between the diabetic group and the control group at rest through the linear indices in the time domain (SDNN and rMSSD) and frequency domain (LF [ms^2^], HF [ms^2^], LF [nu], HF [nu] and LF/HF). Statistically significant differences were observed in the SDNN (p = 0.015), rMSSD (p = 0.015) and LF [ms^2^] (p = 0.031), with lower values for the diabetic group compared to the control group; the HF [ms^2^], LF [nu], HF [nu] and LF/HF presented no significant differences between the groups.

**Table 2:** Values of SDNN, rMSSD, LF [ms^2^], HF [ms^2^], LF [nu] and HF [nu] and LF/HF ratio indices of both groups at rest.

Variables	Control	Diabetes	ANCOVA		
	Mean (SD)		p-value	*Eta-Squared*	*Effect size*
**SDNN**	75.2 (19.8)	50.4 (16.0)	0.015*	**0.200**	**Large**
**rMSSD**	57.6 (18.6)	38.7 (13.3)	0.015*	**0.200**	**Large**
**LF [ms**^**2**^**]**	1874.6 (1392.3)	693.6 (408.1)	0.031*	**0.160**	**Large**
**HF [ms**^**2**^**]**	1222.1 (740.9)	694.9 (405.0)	0.130	0.083	Low
**LF [nu]**	58.2 (15.6)	49.6 (14.0)	0.306	0.039	Low
**HF [nu]**	41.7 (15.6)	50.3 (14.0)	0.307	0.039	Low
**LF/HF**	1.78 (1.17)	1.11 (0.79)	0.275	0.044	Low

ANCOVA = Analysis of Covariance controlled for sex, age, ethnicity, body fat percentage and casual blood glucose; SD = standard deviation; SDNN = standard deviation of all normal RR intervals recorded in an interval of time; rMSSD = root mean square of successive differences between adjacent RR intervals; LF = Low Frequency; HF = high frequency; LF/HF = ratio of low and high frequency.

*p<0.05.

Table 3 (S3, S4, S5 and S6 Files complement) presents the comparisons of the HRV indices in the time domain (SDNN and rMSSD) of the diabetic and control groups at rest and while performing the active orthostatic test. Statistically significant differences were observed for SDNN in relation to group (p = 0.005), while for the rMSSD index, differences were observed in relation to group (p = 0.033), time (p = 0.044) and the interaction between them (p = 0.013). The values were lower during the active orthostatic test in comparison with at rest for both indices.

**Table 3:** Mean values, followed by their standard deviation of the SDNN and rMSSD indices at rest and during the active orthostatic test, adjusted for sex, age, ethnicity, body fat percentage and casual blood glucose.

Variables	Control	Diabetes				
	Mean (SD)		Effect	F	*P*	*Effect size*
**SDNN**			Group	**9.12**	0.005*	**0.253**
M1	75.2 (19.8)	50.4 (16.0)	Time	2.83	0.104	0.095
M2	52.6 (9.5)	37.9 (11.8)	Group x Time	1.35	0.254	0.048
**rMSSD**			Group	**5.04**	0.033*	**0.157**
M1	57.6 (18.6)	38.7 (13.3)	Time	**4.45**	0.044*	**0.141**
M2	26.3 (9.1)	21.0 (12.4)	Group x Time	**7.04**	0.013*	**0.207**

SD = standard deviation; M1 = rest moment; M2 = active orthostatic test moment; SDNN = standard deviation of all normal RR intervals recorded in an interval of time; rMSSD = root mean square of successive differences between adjacent RR intervals.

*p<0.05.

Table 4 (S3, S4, S5 and S6 Files complement) shows the comparison of HRV indices in the frequency domain (LF [ms^2^], HF [ms^2^], HF [nu], LF [nu] and LF/HF ratio) of the diabetic and control groups at rest and while performing the active orthostatic test. A statistically significant difference was observed for LF [ms^2^] in relation to group (p = 0.006), while for the other indices no differences were observed. The values were lower during the active orthostatic test compared with at rest for the indices LF [m^2^], HF [ms^2^] and HF [nu] and higher for the indices LF [nu] and the LF/HF ratio.

**Table 4:** Mean values, followed by their standard deviation, of LF [ms^2^], HF [ms^2^], LF [nu], HF [nu] and LF/HF ratio indices at rest and during the active orthostatic test, adjusted for sex, age, ethnicity, body fat percentage and casual blood glucose.

Variables	Control	Diabetes				
	Mean (SD)		Effect	F	*P*	*Effect size*
**LF [ms**^**2**^**]**			Group	**9.09**	0.006*	**0.252**
M1	1874.6 (1392.3)	693.6 (408.1)	Time	0.295	0.591	0.011
M2	1643.2 (957.5)	693.4 (502.2)	Group x Time	0.099	0.755	0.004
**HF [ms**^**2**^**]**			Group	1.86	0.184	0.065
M1	1222.1 (740.9)	694.9 (405.0)	Time	2.83	0.104	0.095
M2	373.2 (310.4)	317.6 (340.2)	Group x Time	2.55	0.122	0.086
**LF [nu]**			Group	1.02	0.320	0.037
M1	58.2 (15.6)	49.6 (14.0)	Time	0.16	0.693	0.006
M2	80.6 (9.2)	72.2 (15.8)	Group x Time	0.43	0.506	0.016
**HF [nu]**			Group	1.02	0.321	0.037
M1	41.7 (15.6)	50.3 (14.0)	Time	0.16	0.693	0.006
M2	19.3 (9.2)	27.7 (15.8)	Group x Time	0.43	0.515	0.016
**LF/HF**			Group	0.00	0.935	0.000
M1	1.78 (1.1)	1.11 (0.7)	Time	1.52	0.228	0.053
M2	5.8 (4.2)	4.9 (4.4)	Group x Time	0.24	0.626	0.009

SD = standard deviation; M1 = rest moment; M2 = active orthostatic test moment; LF = low frequency; HF = high frequency; LF/HF = ratio of low and high frequency.

*p<0.05.

## Discussion

In the present study the autonomic alterations in children with DM1 and the changes induced by the realization of the active orthostatic test were analyzed. The results showed that children with diabetes presented smaller global HRV and parasympathetic modulation in relation to healthy children and that during the realization of the active orthostatic test, children in both groups demonstrated increased sympathetic activity and reduced parasympathetic activity, and this response was less pronounced in the diabetic group. These changes were independent of sex, age, ethnicity, body fat percentage and casual blood glucose.

The rMSSD index, which represents the parasympathetic activity [7], demonstrated a significant reduction in the group of diabetic children in comparison to the control group, with a great effect size. This result suggests that children with diabetes present a reduction in parasympathetic activity. Although not significant, the HF [ms^2^] index, which is also representative of parasympathetic modulation [7], was reduced in children with DM1. Corroborating these findings, other studies have also shown a reduction in the parasympathetic modulation of children with DM1 [11,13].

Reduced parasympathetic modulation is described in the literature as the first sign of CAN, due to impairment in the long nerve fibers, which damage the vagus nerve that is responsible for about 75% of parasympathetic activity [4,15]. Thus, detecting alterations in this branch of the ANS is extremely important and could help in the prevention and early treatment of the disease [33], guiding patients on appropriate control of diet and physical exercise [12].

In relation to the overall activity of the ANS, the SDNN index, which represents the joint sympathetic and parasympathetic activity [7], was also reduced in the group of diabetic children in comparison to the control group, with an effect size considered as great, suggesting a reduction in the overall activity of the ANS. In accordance with our study, Kardelen et al. [11] evaluated children with DM1 and found a reduction in SDNN values in diabetic children. Özgür et al. [15] found no significant differences when assessing the SDNN index, however, lower values were observed in the group of diabetic children compared to the control. This difference may be related to the methodology used in the study of Özgür et al. [15], who, in addition to not carrying out the proposed adjustments, performed 24-hour analysis using a Holter monitor, which differs from the present study.

The analysis of the LF [ms^2^] index, which enables measurement of the sympathetic performance [7], also demonstrated a significant reduction in the diabetic children, with an effect size considered large, indicating a decrease in sympathetic activity in these children. Studies found in the literature agree with these findings, Kardelen et al. [11] and Chen et al. [13] found a reduction in LF [ms^2^] values in children with DM1, suggesting reduced sympathetic activity.

The data in the frequency domain, transformed into normalized units, contribute to the evaluation of the sympathovagal balance as they represent the percentage that each branch acts out of a total of 100% [7]. The LF and HF indices in normalized units did not present differences, since both the sympathetic activity and parasympathetic were reduced.

Importantly, the observed changes in autonomic modulation were independent of age, sex, ethnicity, body fat percentage and casual blood glucose. It is known that these variables can alter autonomic modulation [15,17–20], therefore, in these children, the disease was predominantly responsible for the observed alterations.

Specifically in relation to the glycemia variable that presented significant differences between the groups, it is believed that hyperglycemia is associated with the start of the pathogenic process and early disease progression [4,23]. Thus, some studies have reported an association between glycemic control and HRV, indicating that early and intensive treatment of glycemic control is a major factor in slowing the progression and preventing the development of CAN [12,15,34].

In relation to the active orthostatic test, the analysis showed statistical significance between group, time and interaction between these factors for the rMSSD index. In both groups a reduction in rMSSD values was observed with the performance of the test, and the deltas of this index between at rest and the active orthostatic test was 31.3 (54.3%) for the control group and 17.7 (45.7%) for the diabetic group. These data indicate that diabetic children have a weaker parasympathetic activity response during the realization of the autonomic test, suggesting abnormal adaptation of ANS to stimuli.

There was also a significant difference between groups for the SDNN index, with a decrease in values with performance of the active orthostatic test in both groups. The deltas between the rest and the test were 22.6 and 12.2 for the control and diabetic groups, respectively, suggesting that diabetic children have a lower reduction in overall HRV compared with healthy children.

In the analysis in the frequency domain, there was a significant difference between the groups only for LF [ms^2^], which is representative of sympathetic activity [7]. The HF [ms^2^] index demonstrated no significant differences between variables. Nevertheless, there was a reduction in the values during the test, suggesting a reduction in parasympathetic activity in both groups.

The indices in the frequency domain in normalized units (LF [nu] and HF [nu]) and the LF/HF ratio demonstrated no statistically significant differences in values. However, there was an increase in LF [nu] and a reduction in HF [nu], as well as an increase in the LF/HF ratio during the realization of the active orthostatic test. These data in the frequency domain suggest that, even without statistically significant differences, there was activation of sympathetic activity in both groups during the realization of the autonomic test.

Only one study, performed by Lucini et al. [14], evaluated performance of the active orthostatic test in diabetic children and adolescents, through indices in the frequency domain. Corroborating the findings of the present study, the authors found that the autonomic test caused sympathetic activation and a reduction in parasympathetic activity; however, these changes were similar in both the diabetic group and the healthy group, which differs from the findings of the present study. This difference may be related to the fact that in the present study, adjustments were carried out by age, sex, ethnicity, body fat percentage and casual blood glucose for all variables.

The cross-sectional design of this study does not allow assessment over time, thus making accompaniment of autonomic changes during the course of the disease impossible, which can be considered a limitation of the study. Furthermore there are controversies in the literature about the alleged predominance of sympathetic activity quantified mainly by LF [33], therefore further studies are needed to confirm the changes in sympathetic activity in this population, and the possible lack of significance in some indices may be related to the sample size.

The fact of the analyzes being adjusted for sex, age, ethnicity, body fat percentage and casual blood glucose, factors that alter autonomic modulation and which were not used in other studies, in addition to the association between HRV and the active orthostatic test, a technique that has been shown to be effective for analysis of the autonomic behavior of this population, must be considered strong points of the study.

The results show that diabetic children, regardless of age, sex, ethnicity, body fat percentage and casual blood glucose, present autonomic changes characterized by a reduction in the overall variability and parasympathetic activity and that the realization of the active orthostatic test promoted an increase in sympathetic modulation and reduced parasympathetic modulation in children of both groups, and that this response was less pronounced in diabetic children.

In summary, it is understood that autonomic alterations are present in the early stages of DM1; this fact makes prevention and early treatment important in order to restore the activity of the ANS, avoiding complications arising from these changes and improving the prognosis of the disease and the quality of life of these patients [34].

## Supporting Information

S1 File Characteristics of the diabetics children. (XLS)

S2 File Characteristics of the healthy children. (XLS)

S3 File Values of SDNN, rMSSD, LF [ms^2^], HF [ms^2^], LF [nu] and HF [nu] and LF/HF ratio indices of diabetic group at rest. (XLSX)

S4 File Values of SDNN, rMSSD, LF [ms^2^], HF [ms^2^], LF [nu] and HF [nu] and LF/HF ratio indices of healthy group at rest. (XLS)

S5 File Values of SDNN, rMSSD, LF [ms^2^], HF [ms^2^], LF [nu] and HF [nu] and LF/HF ratio indices of diabetic group during the active orthostatic test. (XLS)

S6 File Values of SDNN, rMSSD, LF [ms^2^], HF [ms^2^], LF [nu] and HF [nu] and LF/HF ratio indices of healthy group during the active orthostatic test. (XLS)
